# Supplementary material for: Yiai Fuzheng decoction inhibits triple-negative breast cancer by remodeling the immune microenvironment
Source: Front Immunol. 2025 Sep 30;16:1615631. doi: 10.3389/fimmu.2025.1615631 (PMC12518410; doi:10.3389/fimmu.2025.1615631)
Supplement: Supplementary file 2 [file DataSheet1.zip › Supplementary File 2/Treatment/C_vs_D_vs_H/06_Pathway_Analysis/Pathway_Analysis/Sig_Pathways_ID/Starch and sucrose metabolism.pdf]

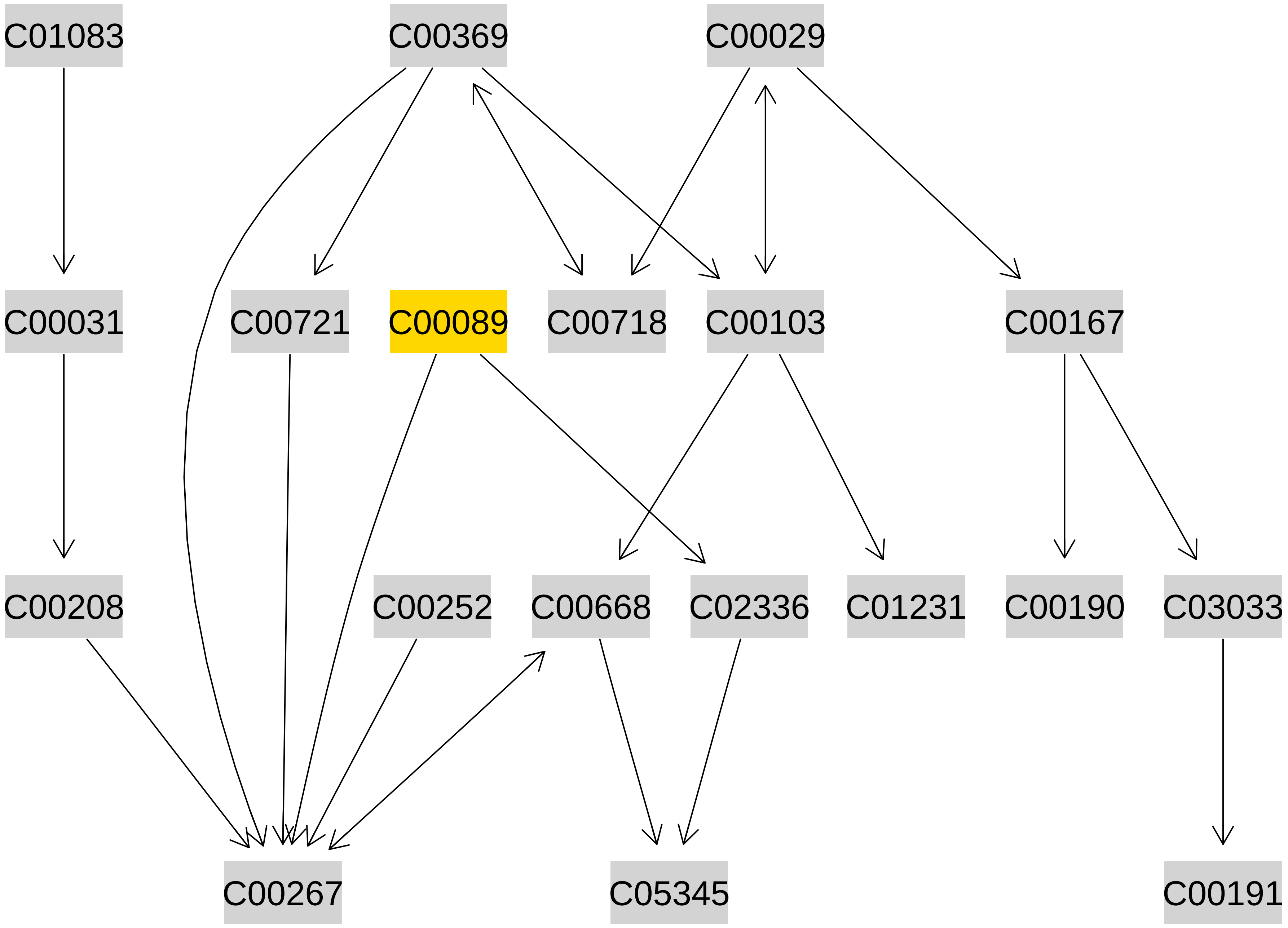

C01083
C00369
C00029
C00031
C00721
C00089
C00718
C00103
C00167
C00208
C00252
C00668
C02336
C01231
C00190
C03033
C00267
C05345
C00191
